# Isolated primary craniopharyngioma of the fourth ventricle: a rare case report and literature update

**DOI:** 10.3389/fonc.2026.1663541

**Published:** 2026-01-29

**Authors:** Ligang Chen, Xinyu Yang, Xuantong Liu, Geyu Wang, Xinning Li, Xiaoyu Sun, Sizhe Feng, Guobiao Liang

**Affiliations:** 1 Department of Neurosurgery, General Hospital of Northern Theater Command, Shenyang, China; 2 Department of Pathology, General Hospital of Northern Theater Command, Shenyang, China

**Keywords:** craniopharyngioma, the fourth ventricle, surgical resection, pathology, case report

## Abstract

**Background:**

Craniopharyngiomas usually occur in the saddle and suprasellar regions, and primary cases occurring in the fourth ventricle are extremely rare, with only eight definite cases reported in the English literature. In this article, we present the ninth case of primary craniopharyngioma of the fourth ventricle.

**Case description:**

The patient was a 51-year-old man who complained of progressive occipital headache, unsteady gait and vomiting. Imaging showed a cystic occupying lesion in the fourth ventricle with calcification of the cystic wall, which compressed the aqueduct and led to obstructive hydrocephalus. Images of the saddle and suprasaddle regions were completely normal, and there was no history of surgery. The patient underwent suboccipital median approach, cerebellar hyaloid surgery, and total resection of the tumor. Pathology suggested an adamantinomatous type craniopharyngioma with positive β-catenin nuclei. Postoperative recovery was good, and no recurrence was seen at 12-month follow-up.

**Conclusion:**

The case in this article is an isolated primary fourth ventricle craniopharyngioma with no history of saddle region extension or surgery. Its clinical presentation and pathologic features support an embryologic mechanism of residual development of Rathke’s capsule. It is recommended that craniopharyngiomas be included in the differential diagnosis when evaluating locoregional lesions in the posterior cranial fossa, especially if the imaging manifestations are cystic or contain calcifications.

## Introduction

Craniopharyngioma is a rare benign epithelial tumor that accounts for approximately 1.2%-4.6% of all intracranial tumors (1). It originates from the embryonic remnants of the Rathke’s capsule, is usually located in the saddle or suprasellar region, is slow-growing, cystic or mixed cystic-solid, and may compress important structures such as the optic cross, hypothalamus, pituitary gland, or third ventricle (2). Although craniopharyngiomas can occur in atypical sites such as the infrasellar region, anterior cranial fossa, temporal lobe, or cerebellopontine angle, primary occurrence in the fourth ventricle is extremely rare (3, 4). Some cases result from extension of the tumor from the sellar region to the posterior cranial fossa, while others are postoperative implantation recurrences (5). In contrast, isolated, non-extending, non-surgical history of primary craniopharyngioma of the fourth ventricle constitutes an extremely rare ectopic subtype (6). Up to now, eight cases of definite primary craniopharyngioma of the fourth ventricle have been reported in the English literature, all meeting the following criteria: no history of sellar region involvement, normal MRI findings in the sellar region, lesion located in the fourth ventricle, and pathological confirmation of craniopharyngioma (6, 7). The patient reported in this paper was a 51-year-old male with clinical presentation and imaging features highly consistent with previous reports: no history of surgery, no sellar region lesion, and pathologically confirmed adamantinomatous type craniopharyngioma. This paper may represent the ninth case of primary fourth ventricle craniopharyngioma in the literature. With this case, we aim to add to the literature, emphasize its embryonic developmental mechanism, and reinforce clinical awareness of the recognition and diagnosis of this pathotype (7).

## Case description

The patient was a 51-year-old male with no history of intracranial disease or surgery. He was admitted to the hospital with “progressive headache, recurrent nausea and vomiting”, which lasted for about two months. There were no positive findings on physical examination, normal neurological examination, normal muscle strength of both lower limbs, and no visual or hearing impairment. Vital signs were stable at the time of admission, with a Glasgow Coma Score of 15. The pupils were equal in size and round, 3 mm in diameter, and responsive to light. Mini-Mental State Examination score was 28.

### Imaging evaluation

**Figure 1:**
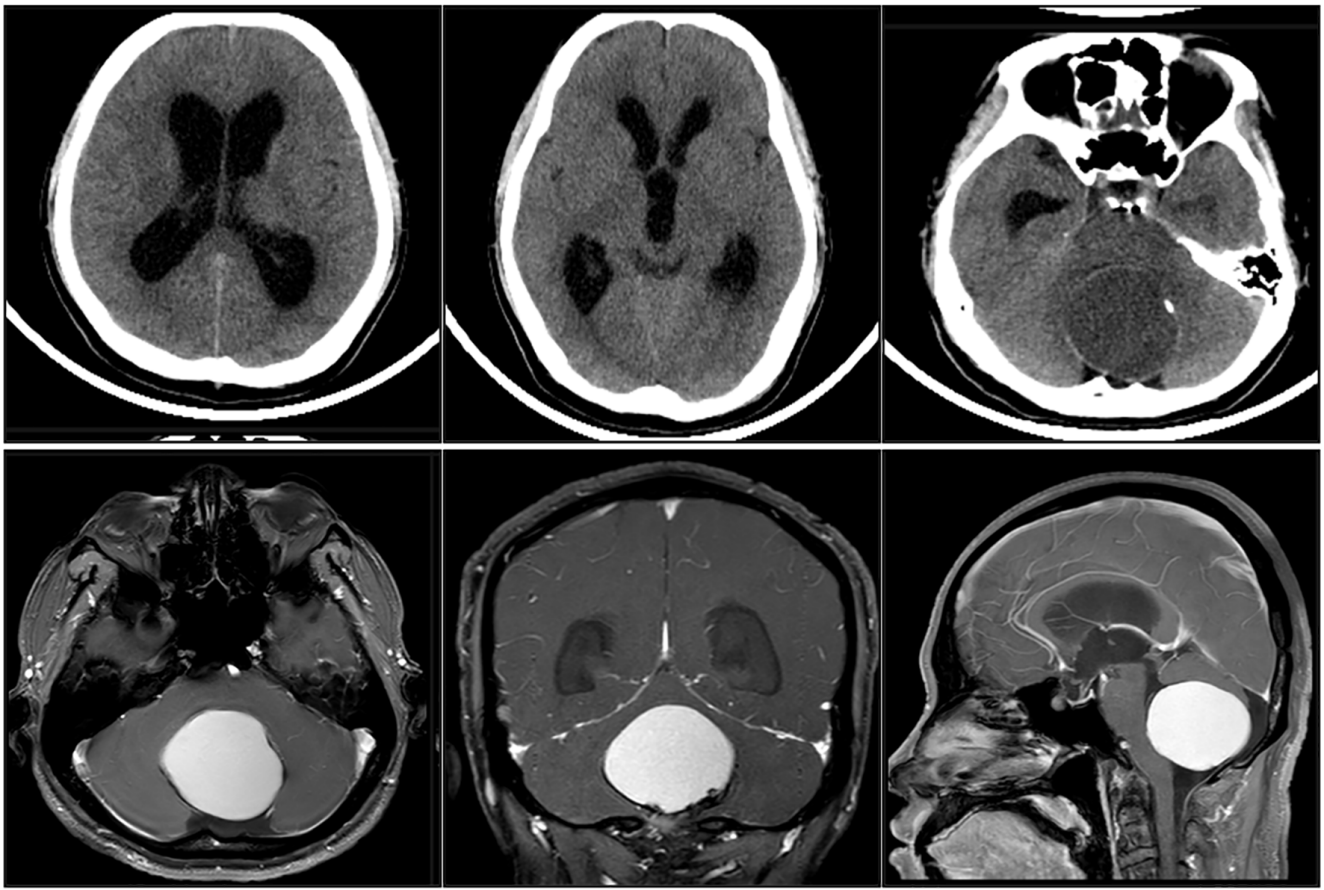
Admission cranial CT examination.

After admission, cranial CT examination showed a low-density round-like occupying lesion in the fourth ventricle, with surrounding calcification, compression of the aqueduct, symmetrical enlargement of the third ventricle and lateral ventricles, suggesting obstructive hydrocephalus (**Figure 1**). MRI examination showed a round-like cystic tumor measuring approximately 4.8 cm × 4.8 cm × 4.0 cm in the fourth ventricle (**Figure 1**). The cystic portion of the lesion showed slightly high signal in T1-weighted image and obvious high signal in T2-weighted image. The cystic portion of the lesion was slightly high signal in T1-weighted image, and it was obviously high signal in T2-weighted image, and there was no obvious enhancement in the enhanced scan, and the fourth ventricle was narrowed and fissured by the compression. There was no extension of the lesion to the saddle region or suprasellar region, and the anatomy of the pituitary stalk, pituitary proper and optic cross was clear with normal signal (**Figure 1**). There was no intracranial multifocal lesion, and no supratentorial area occupation was seen. Combined with the imaging manifestations, cystic tumor of the fourth ventricle was initially considered, and further differential diagnosis was needed. Preoperatively, we mainly considered ventricular meningioma, intraventricular epidermoid cyst or atypical glioma and ectopic craniopharyngioma.

### Surgical procedure

**Figure 2:**
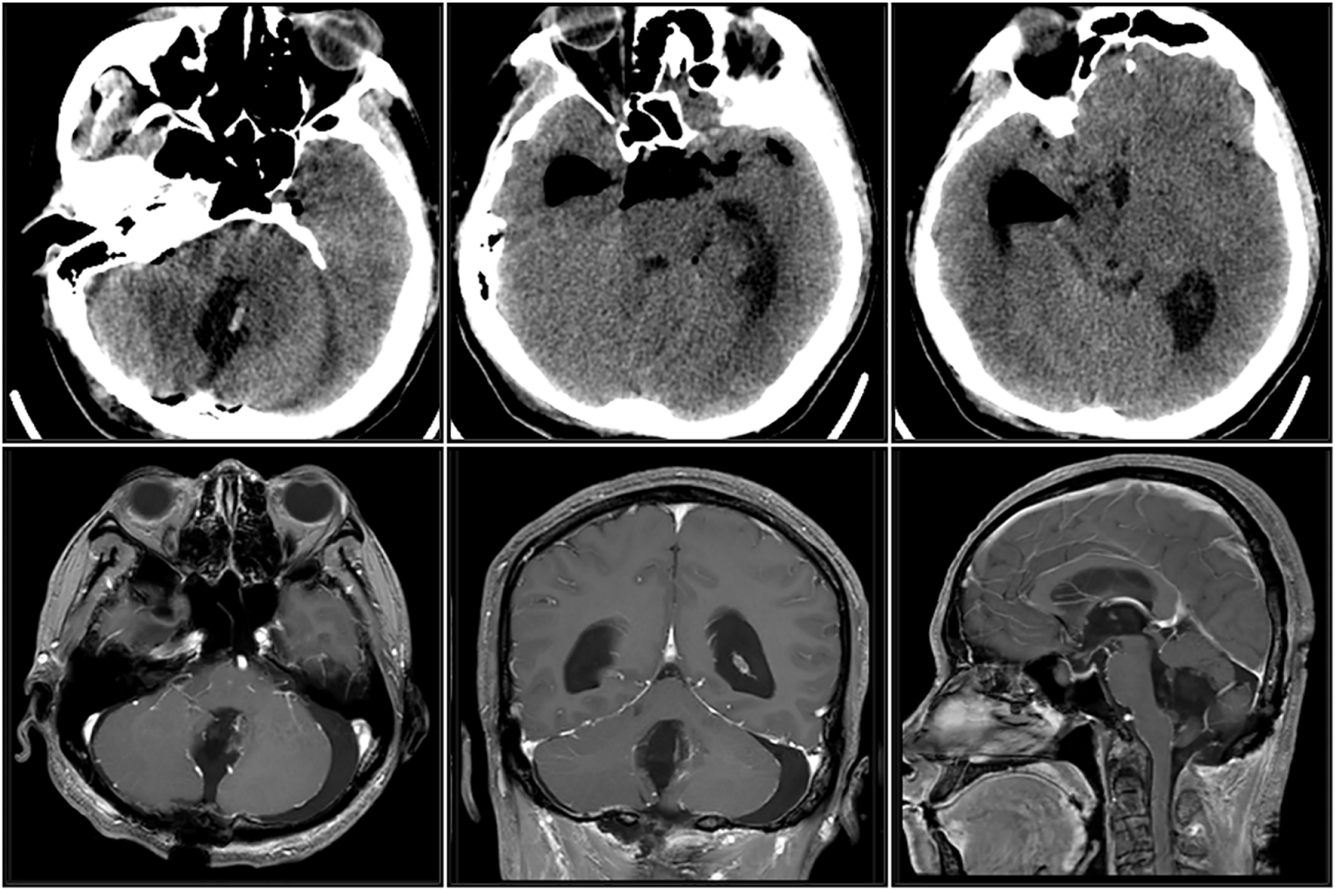
Postoperative CT and MRI.

The patient was placed in the prone position under general anesthesia, and a median suboccipital craniotomy was performed. Under the microscope, the fourth ventricle was entered through the cerebellar hiatus, and the tumor was revealed after opening the cerebellar plexus. The lesion was located in the cavity of the fourth ventricle, and the cystic part was obviously tense. Firstly, we punctured and aspirated the “brown greasy fluid”, and the shiny crystals could be seen under the microscope. The cystic wall was separated from the solid part and resected. The cystic wall tumor was gray, tough, and partially adhered to the tissue at the base of the fourth ventricle, with clear boundaries. On the basis of fully protecting the medulla oblongata and the conduit orifices, the entire lesion was gradually resected. No obvious hemorrhage or brainstem injury was observed during the operation. Postoperative CT and MRI showed that the tumor had been completely resected and the fourth ventricle was well reconstructed (**Figure 2**). After surgery, the patient awoke well, with no new neurological deficits, and his gait returned to normal. No hydrocephalus was found in the repeat cranial CT, and the external drain was removed on the 4th day after surgery.

### Pathologic examination

**Figure 3:**
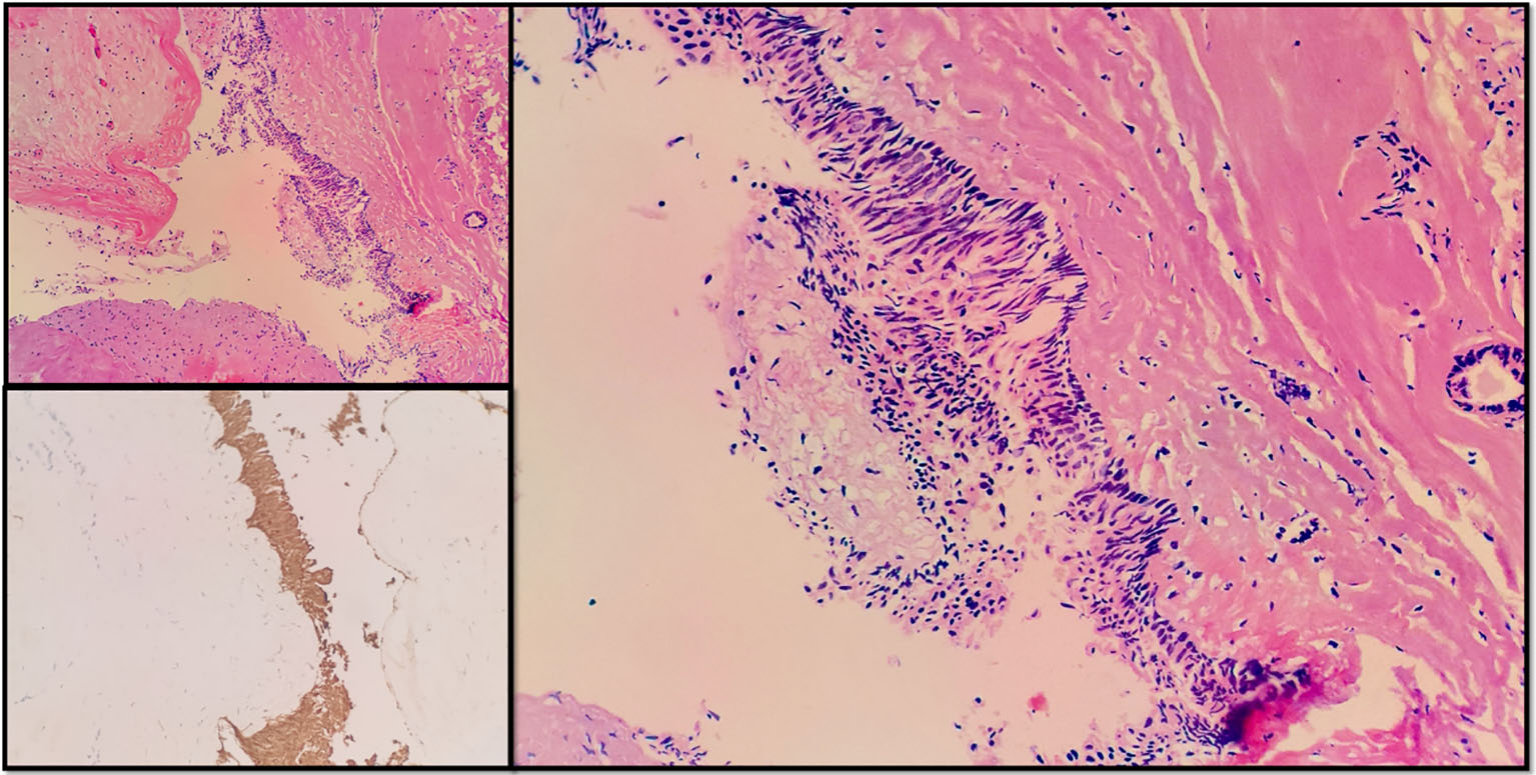
Immunohistochemistry.

Intraoperative samples were taken and sent for pathologic examination; microscopically, basal fenestrated arrangement of squamous cells with papillary growths, interstitial laxity, and abundant vascularity were seen. Abundant wet keratin deposition, focal calcification and cholesterol clefts were seen. Immunohistochemistry suggested CK-P (+) and strong nuclear positivity of β-catenin cells, which supported adamantinomatous type of craniopharyngioma (**Figure 3**). The Ki-67 proliferation index was about 10%, and there was no anisotropy or active mitosis.

### Follow-up

The patients recovered well after surgery without complications such as low sodium or pituitary dysfunction. No tumor recurrence was seen in the 3-month postoperative review. The patient’s life status is good and her neurological function score is perfect, she has resumed her normal life and work.

## Discussion and literature review

We reviewed eight cases of primary fourth ventricle craniopharyngioma published between 1996 and 2023, along with the case reported herein, as detailed in **Table 1**.

**Table 1:** Reported cases of isolated 4 ventricle craniopharyngioma to date.

Author	Age/Sex	Presenting symptoms and signs	Tumor size (cm)/Cystic (C) or Solid (S)	Surgical approach	Histological type	Outcome and follow-up
E. M. BASHIR et al. (27)	23/M	Headache, ataxia, bilateral papilledema	Not mentioned/C	Suboccipital Retrosigmoid Approach	craniopharyngioma	uneventful and neurological recovery fully
Gopal B. Shah et al. (28)	12/F	Headache, left abducent palsy	Not mentioned/C	Tonsillovellar approach	craniopharyngioma	uneventful and neurological recovery fully
Andres H. Pena et al. (5)	20/M	Headache, Gardner syndrome	1.7cm/C	Transvermian Approach	adamantinomatous craniopharyngioma	no evidence of residual or recurrent tumor after 3 months
Juan Antonio Álvarez Salgado et al. (29)	29/M	Right facial paralysis, diplopia, Gardner syndrome	2cm/C	Skull base approach	Craniopharyngioma (including keratocystic variant)	Not mentioned
Abdulhadi Y et al. (2)	24/M	Headache, trouble walking, diplopia	7.2cm×5.5cm×4.1cm/C	posterior fossa craniectomy	adamantinomatous type craniopharyngioma	Asymptomatic and no evidence of a recurrence after 2−year follow−up.
Hiroya Uemura et al. (6)	63/F	Headache	3.2cm/c	midline suboccipital craniotomy	acquired cerebral pontine glioma	no evidence of tumor recurrence in 2-month follow-up
Nazmin Ahmed et al. (7)	16/M	Headache, trouble walking	4.5×3.5 cm/S	telovelar approach	craniopharyngioma of the adamantinomatous type	no evidence of tumor recurrence in 1-year follow-up
Can Du et al. (30)	4/M	discovered during follow-up examination	Not mentioned/C	right subfrontal craniotomy	adamantinous craniopharyngioma	disease free since Surgical treatment
Ligang Chen et al.	51/M	Progressive headache, recurrent nausea and vomiting	4.8 cm×4.8cm×4.0 cm/C	median suboccipital craniotomy	adamantinomatous type of craniopharyngioma	recovered well and no evidence of tumor recurrence in 3-month follow-up

Craniopharyngioma is a benign epithelial tumor derived from the remnants of Rathke’s cystic embryo, which usually occurs in the saddle and suprasellar regions, and its clinical presentation is mainly related to the tumor’s compression on the optic nerve, the hypothalamus, and pituitary function. Typical cases tend to present with decreased visual acuity, endocrine disorders and increased intracranial pressure (2, 8). Ectopic craniopharyngiomas, especially those originating in the fourth ventricle, are extremely rare and often difficult to diagnose preoperatively due to nonspecific symptoms and lack of typical imaging features (5–7).

### Pathogenesis

Craniopharyngiomas typically originate in the sella turcica region, but although rare, ectopic occurrences have been documented in the literature. These ectopic craniopharyngiomas may be found in locations such as the clivus, nasopharynx, third ventricle, cerebellopontine angle, and fourth ventricle (9). Regarding the formation mechanism of primary craniopharyngiomas of the fourth ventricle, the commonly accepted hypothesis is the embryonic developmental abnormality theory, i.e., during upward migration of the Rathke’s capsule, some of the epithelial cells are “lost” and remain in the subcapsular region, which later evolve into tumors (7). The epithelial chemotaxis hypothesis has also been proposed, in which neuroepithelial cells undergo chemotaxis in response to certain stimuli to form squamous epithelium, which in turn becomes tumorigenic (2). However, the latter has limited evidence and has not been widely supported (8). In our case, there was no history of any tumor in the saddle region or previous neurosurgery, and MRI showed clear anatomy and normal signal in the saddle region and suprasellar region, with no tumor extension, which clearly supported the diagnosis of “primary isolated fourth ventricle craniopharyngioma” (6). This is highly consistent with the case reported by Ahmed et al. in 2023 (4).

### Imaging features and differentiation

Typical craniopharyngiomas are imaged as mixed cystic and solid, with cystic fluid often high signal on T1-weighted images and high signal on T2-weighted images, and foci of calcification are clearly visible on CT (10). However, in cases of ectopic fourth ventricle, the imaging features are often atypical. Most cases in the literature have shown solid or inhomogeneous enhancement, with less cystic degeneration, which can be easily misdiagnosed as ventricular meningioma, astrocytoma, and intraventricular cyst (11). In this case, the image showed a cystic lesion within the fourth ventricle, and the calcified foci were clearly visible on CT, with slightly high signal in T1 and obviously high signal in T2, and no obvious enhancement was seen in the enhancement scan, suggesting that the inner part of the lesion contained high-protein or lipid components, which was close to the typical performance of craniopharyngiomas in saddle region (12). However, due to its special location, craniopharyngioma was not listed as the first choice of diagnosis before surgery, suggesting that the sensitivity of ectopic lesions should be improved in clinical work (13).

### Pathologic type and biological behavior

The pathology of this case was adamantinomatous type of craniopharyngioma with features including nests of squamous epithelial cells, stellate reticulation, wet keratins, and cholesterol clefts, positive immunohistochemical nuclei for β-catenin, and a low Ki-67 index (14, 15). This subtype is common in children and adolescents, but is also seen in adults and accounts for the majority of reported fourth ventricle cases (16, 17). In contrast, the papillary type tends to be adult-onset, with predominantly solid enhancement on the image, often accompanied by the BRAF V600E mutation; however, the papillary subtype is not present in all current reports of fourth ventricles (18).

### Surgical strategy and prognosis

The choice of surgical route for posterior cranial fossa tumors is very critical, and the suboccipital median approach combined with the cerebellar pallidum approach is the current standard strategy for the management of tumors in the fourth ventricle, which can protect the brainstem and cerebellar functional areas while fully exposing the tumor (19). In this case, complete resection was successfully achieved intraoperatively, and the postoperative recovery was good, suggesting high surgical safety and feasibility. It has been reported in the literature that if the tumor can be completely resected, the prognosis is usually good; if it is incompletely resected, the risk of recurrence is significantly increased, and it is recommended to cooperate with fractionated stereotactic radiotherapy (FSRT) or Gamma Knife therapy in the postoperative period (20, 21). There is no clear consensus in the current literature recommending whether ectopic craniopharyngiomas should routinely receive radiotherapy, but recurrences are mostly associated with intraoperative residuals (22).

Modern craniopharyngioma management emphasizes multimodal therapy and individualized strategies. International guidelines, such as those from the European Association of Neurological Surgeons (EANS) (23), emphasize balancing long-term tumor control with preserving patient quality of life, particularly hypothalamic function. Surgical resection remains the primary initial treatment modality, with the choice between endoscopic transnasal approaches or craniotomy determined by tumor origin, subtype, and extent of extension. For tumors with significant adhesion to critical structures like the hypothalamus, current trends favor avoiding aggressive radical resection to reduce severe complication risks. Instead, a strategy of surgical debulking followed by postoperative radiotherapy is increasingly adopted. Additionally, targeted drug therapies (e.g., BRAF/MEK inhibitors) offer new hope for recurrent or refractory cases (24).

### Literature summary

As of 2024, eight cases of primary fourth ventricular craniopharyngiomas have been definitively reported, all without saddle extension or surgical history, and with a predominantly adamantinomatous type of pathology (22). The present case may be the ninth in the literature. A review of the literature suggests that this type of lesion, although rare, is not incidental and should be considered as one of the differential diagnoses for occupying lesions in the posterior cranial fossa, especially when imaging suggests mixed cystic-solid structures, lipoid-containing components, or calcifications (25, 26).

## Conclusions

We report a rare case of isolated primary craniopharyngioma of the fourth ventricle with clinical and imaging presentation highly consistent with previous reports in the literature. The diagnosis of this case further supports the theory of abnormal embryonic development leading to ectopic residual Rathke’s capsule cells. Surgery is the mainstay of treatment for these lesions, and the prognosis is usually favorable after complete resection. We suggest that craniopharyngioma should be included in the differential diagnosis when evaluating tumors of the posterior cranial fossa, especially cystic solid lesions of the fourth ventricle, to improve early recognition and guide a rational surgical strategy.

## Data Availability

The raw data supporting the conclusions of this article will be made available by the authors, without undue reservation.
